# Fine‐Needle Aspirate Cytology of Feline Pulmonary Langerhans Cell Histiocytosis in Two Cats

**DOI:** 10.1111/vcp.70141

**Published:** 2026-08-14

**Authors:** Karuna Katariwala, Martim Matias, Isabel Amores‐Fuster, Doroteia Bota, Guido Rocchigiani, Carolina Naranjo, Nazaré Pinto da Cunha, Martina Piviani

**Affiliations:** ^1^ Department of Clinical Sciences and Advanced Medicine, School of Veterinary Medicine University of Pennsylvania Philadelphia Pennsylvania USA; ^2^ Faculty of Veterinary Medicine–Lusófona University–Lisbon University Center Lisbon Portugal; ^3^ Department of Small Animal Clinical Sciences, Institute of Veterinary Science University of Liverpool Neston UK; ^4^ Anicura Restelo Veterinary Hospital Lisbon Portugal; ^5^ Department of Veterinary Anatomy, Physiology and Pathology, Institute of Infection, Veterinary and Ecological Sciences, Leahurst Campus University of Liverpool Neston UK; ^6^ IDEXX Laboratories Barcelona Spain; ^7^ Vedis – Veterinary Expertise in Diagnosis Porto Portugal

**Keywords:** Birbeck granules, CD204, e‐cadherin, histiocytes, iba‐1, lung cytology, transmission electron microscopy

## Abstract

Feline pulmonary Langerhans cell histiocytosis (FPLCH) is a rare histiocytic disorder in cats, usually diagnosed post‐mortem following natural death or euthanasia for severe respiratory distress. Antemortem diagnosis has been reported only once, achieved by cytologic examination of bronchoalveolar lavage fluid combined with immunocytochemistry, whereas fine‐needle aspiration (FNA) of lung nodules was previously reported as non‐diagnostic. Here we describe two cases of FPLCH in which cytology of ultrasound‐guided lung‐nodule FNA yielded diagnostic material, including from one cat with an atypical clinical presentation. Whereas case 1 presented with severe respiratory distress, case 2 had no respiratory signs. Both specimens were highly cellular and composed of atypical cells morphologically most consistent with histiocytes showing moderate atypia. A combination of postmortem gross, histopathologic, immunophenotypic, and ultrastructural findings confirmed the cytologic suspicion of FPLCH. To our knowledge, this is the first cytologic description of FPLCH in FNA samples from lung nodules and the first report of a confirmed case without respiratory signs.

## 1 Case 1

An 8‐year‐old castrated male European Shorthair cat presented to a Veterinary Hospital for investigation of a 1‐year history of progressive weight loss with recently increased respiratory rate and cough. Clinical examination revealed tachypnea, and the body condition score was slightly low (3/9). Hematology and biochemistry results had no major abnormalities. Total T4 was within normal limits.

Thoracic radiographs had a multifocal alveolar pattern with areas of lung consolidation (Figure 1A,B). On bronchoscopy, the mucosal surface was irregular. Initially, bronchoalveolar lavage was performed, and samples were submitted for cytologic examination and microbiological culture. Cytology results were compatible with pyogranulomatous inflammation, and microbiology analysis was negative for both bacterial and fungal agents (Agar MacConkey, 37°C, Aerobic incubation, 48 h; Blood Agar, 37°C, Aerobic incubation, 48 h; Agar Sabouraud chloramphenicol, 37°C, Aerobic conditions, 48 h; Brain‐Heart Infusion enrichment).

**FIGURE 1:**
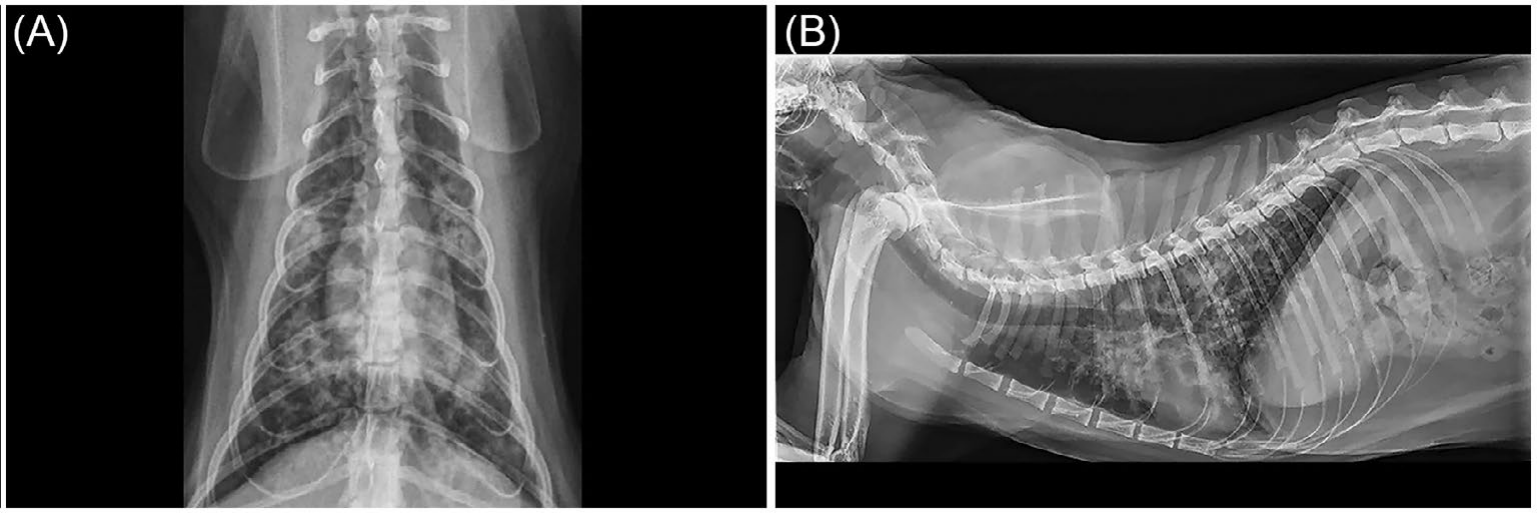
Thoracic radiographs of case 1. (A) Ventro‐dorsal view. (B) Left lateral view. The lungs have a multifocally distributed alveolar pattern with coalescing areas, along with areas of increased soft tissue opacity (consolidation).

A computed tomography (CT) scan had significant bilateral consolidations primarily located around the peri‐hilar and peribronchovascular regions, with some exhibiting peripheral cavitation. Certain distal bronchi appeared tortuous, suggesting either atelectasis or anatomic distortion. Additionally, there were multifocal nodules primarily distributed along the peribronchovascular areas, suggestive of chronic airspace disease with diffuse distribution.

Oral and inhaled corticosteroids were used initially, which briefly improved the cat's clinical condition, decreasing his respiratory rate and cough episodes for 2 months, after which hyporexia, weight loss, and worsening of the lung pattern on thoracic radiographs were noted in a follow‐up appointment.

An ultrasound‐guided fine‐needle aspiration (FNA) of the lung (Figure 2) revealed a population of round to oval, medium‐ to large‐sized cells, with a variable amount of pale to light blue cytoplasm, which rarely contained a few dark blue granules, and irregular and poorly distinct borders. Nuclei were round to oval with coarse to clumped chromatin and inconspicuous nucleoli. Cytologic atypia was moderate and included moderate anisocytosis and anisokaryosis, rare binucleation and multinucleation, rare macrocytosis, and occasional atypical mitotic figures. These findings were highly suspicious for a malignant neoplastic process, with the differential diagnosis including feline pulmonary Langerhans cell histiocytosis (FPLCH), amelanotic melanoma, undifferentiated sarcoma, and histiocytic sarcoma (HS). FPLCH was prioritized over histiocytic sarcoma, as the latter typically exhibits a higher degree of cytologic atypia. As an inflammatory granulomatous condition could not be completely excluded, Ziehl‐Neelsen and Periodic‐acid Schiff (PAS) stains were applied to the previously stained slides, but no organisms were seen.

**FIGURE 2:**
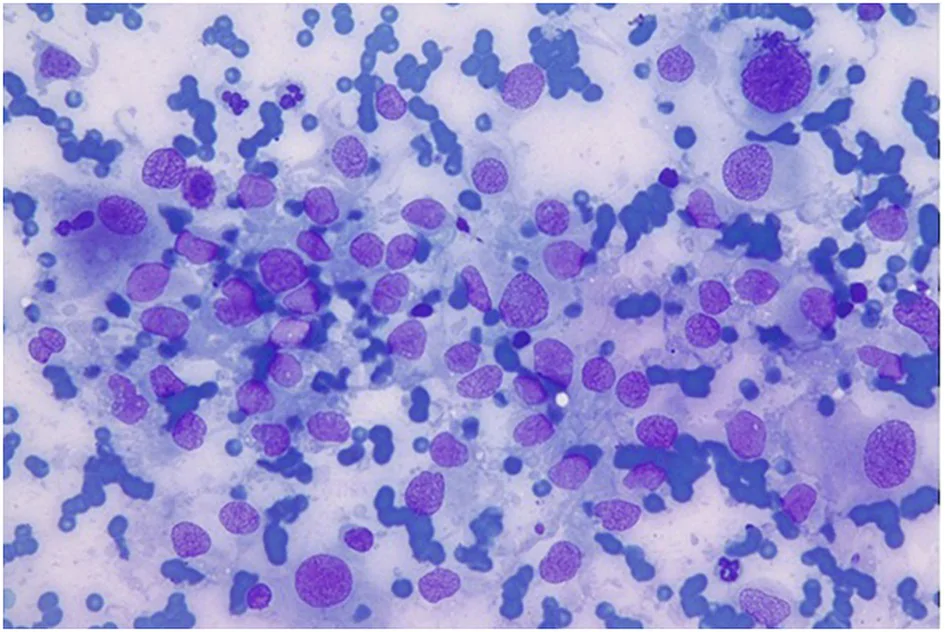
Fine‐needle aspirate cytology of lung nodules from case 1. Monomorphic atypical cells were oval to spindle to stellate shaped with indistinct cytoplasmic edges. Cytoplasm was moderate in quantity and light blue. Nuclei were round to bean‐shaped with moderate indentation, reticular chromatin, and one to three inconspicuous nucleoli. Anisocytosis and anisokaryosis were moderate. Diff‐Quik stain, 400×.

A tyrosine kinase inhibitor (TKI) was introduced therapeutically. Respiratory signs were milder over the following month, but weight loss and anorexia ensued, and the clinical condition abruptly worsened over the course of 1 week. The cat developed respiratory insufficiency and died at home.

Lung samples were collected post‐mortem, fixed in formalin, and submitted for histopathological examination (Figure 3A–C). Eighty percent of the lung parenchyma was occupied and replaced by an expansile population of round to plump spindle cells arranged in solid sheets, obliterating airways and associated with a sparse supporting stroma. These cells had indistinct borders, moderately fibrillar to vacuolated eosinophilic cytoplasm, oval nuclei with finely punctate chromatin, and a small, round, basophilic central nucleolus. Occasional cells had an indented nucleus and a more prominent nucleolus. Anisocytosis and anisokaryosis were moderate, with occasional karyomegalic cells and occasional multinucleation. Nine mitotic figures were identified in 2.37 mm^2^. Few admixed lymphocytes, plasma cells and hemosiderophages were also present.

**FIGURE 3:**
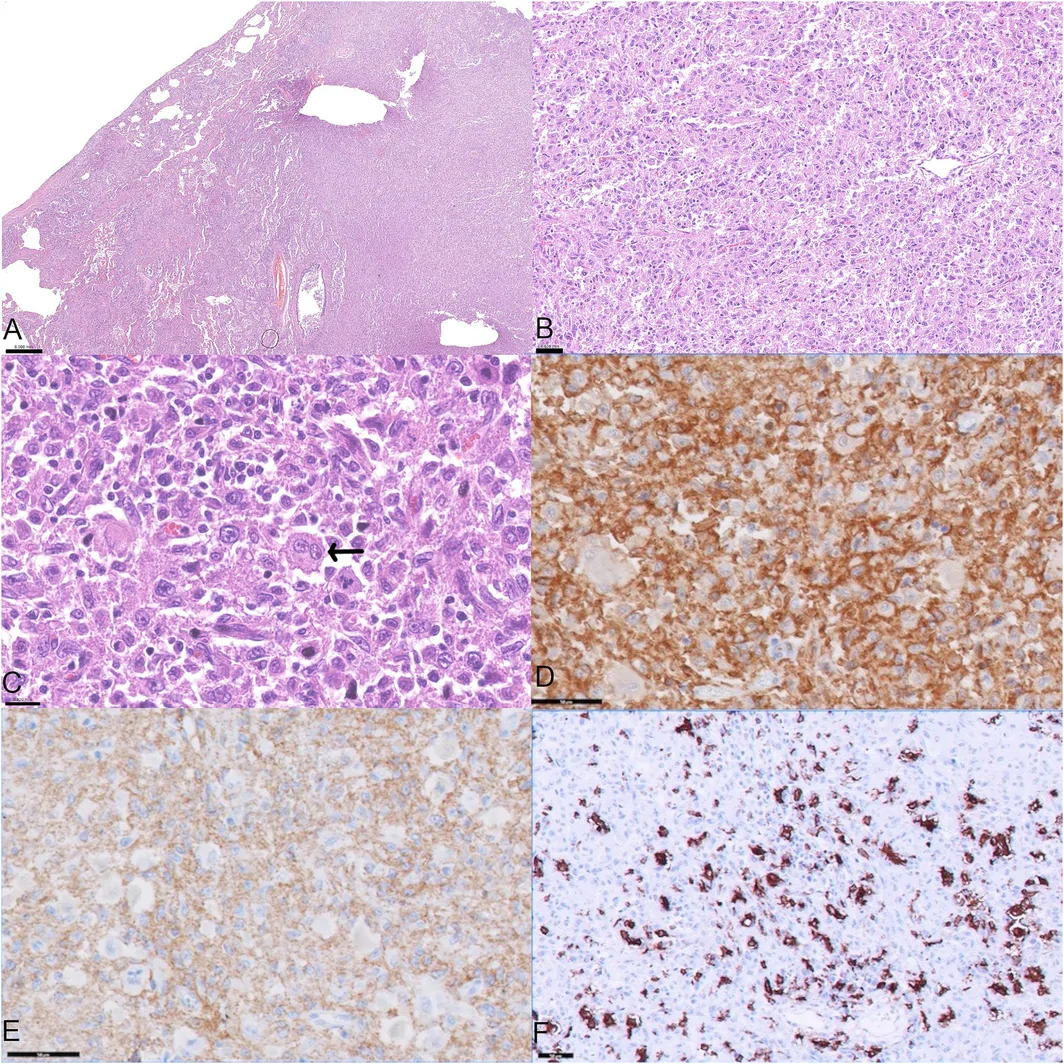
Histological and immunohistochemical features of case 1. (A) There are nodular, poorly demarcated, densely cellular, infiltrative neoplastic masses effacing most of the alveolar parenchyma and, to a lesser extent, the pleura. H&E. Bar = 0.5 mm. (B) The neoplastic cell population is composed of solid sheets of round cells filling alveolar spaces and distorting the pulmonary architecture. H&E. Bar = 50 μm. (C) Neoplastic cells are round to oval and have a moderate amount of eosinophilic, occasionally vacuolated cytoplasm and oval nuclei with finely punctate chromatin, and a small, round, basophilic central nucleolus. Anisocytosis and anisokaryosis are moderate, with occasional slightly karyomegalic cells and occasional multinucleation (arrow). H&E. Bar = 20 μm. (D) Atypical histiocytes have strong anti‐Iba1 membranous and cytoplasmic labeling. IBA‐1 immunohistochemistry (IHC). Hematoxylin counterstain. Bar = 20 μm. (E) Moderate membranous and cytoplasmic granular reactivity to anti‐E‐Cadherin. E‐cadherin IHC. Hematoxylin counterstain. Bar = 20 μm. (F) Approximately 20% of the cells obliterating lung parenchyma are CD204 positive and are interpreted as associated inflammatory macrophages. The neoplastic population is CD204 negative. CD204 IHC. Hematoxylin counterstain. Bar = 20 μm.

An immunohistochemistry panel of anti‐Iba1, anti‐CD204 and anti‐E‐Cadherin (Table S1) was performed on additional sections of the same lung tissue to further characterize the proliferative lesions (Figure 3D,F). There was strong anti‐Iba1 membranous and cytoplasmic labeling in more than 90% of the infiltrating cells (Figure 3D). Moderate membranous and cytoplasmic granular reactivity to anti‐E‐Cadherin in approximately 80% of the infiltrating cells was seen (Figure 3E). Robust cytoplasmic reactivity to anti‐CD204 was observed in approximately 20% of the cells scattered among the atypical cells obliterating terminal airways (Figure 3F). These CD204‐positive cells were interpreted as resident macrophages, similar to those filling the surrounding alveolar spaces.

## 2 Case 2

A 10‐year‐old spayed female domestic shorthaired cat was referred to a Small Animal Teaching Hospital's oncology department for evaluation of a suspected intestinal tumor. The cat experienced progressive weight loss over 4–5 weeks and had become hyporexic in the week prior to referral.

On presentation, the cat was quiet and responsive and in lean body condition. The mucous membranes were slightly pale, and the patient was approximately 5% dehydrated. Thoracic auscultation revealed slightly increased respiratory sounds in all lung fields, with a respiratory rate of 48 breaths per minute and normal effort. No appreciable peripheral lymphadenopathy was noted. Abdominal palpation revealed a thickened mid‐abdominal region, and palpation was resented.

A complete blood cell count revealed lymphopenia (0.54 × 10^9^/L, RI: 1.5–7.0). The biochemistry profile included elevated liver enzymes (ALP 109 U/L, RI: 0–40; ALT 187 U/L, RI: 0–50). Blood glucose measured with a handheld glucometer was elevated (13.9 mmol/L, RI: 3.9–8.0). Urinalysis from a cystocentesis sample revealed mild hematuria (5–7 RBC/HPF) and proteinuria (2+).

Abdominal ultrasound demonstrated a mildly hyperechoic liver and splenomegaly, consistent with the thickened area palpated. Ultrasound‐guided FNA of the spleen and liver revealed extramedullary hematopoiesis and mild mixed hepatocellular vacuolar changes (mild non‐lipid vacuolar change with minimal lipid accumulation), respectively.

Thoracic radiographs demonstrated a diffuse bronchointerstitial pattern with focal areas of increased soft tissue opacity, which were interpreted as nodules (Figure 4A,B). Bronchoscopy identified a small amount of mucus in the major airways. FNAs from the lung nodules and bronchoalveolar lavage fluid (BALF) were collected and stained with modified Wright's stain.

**FIGURE 4:**
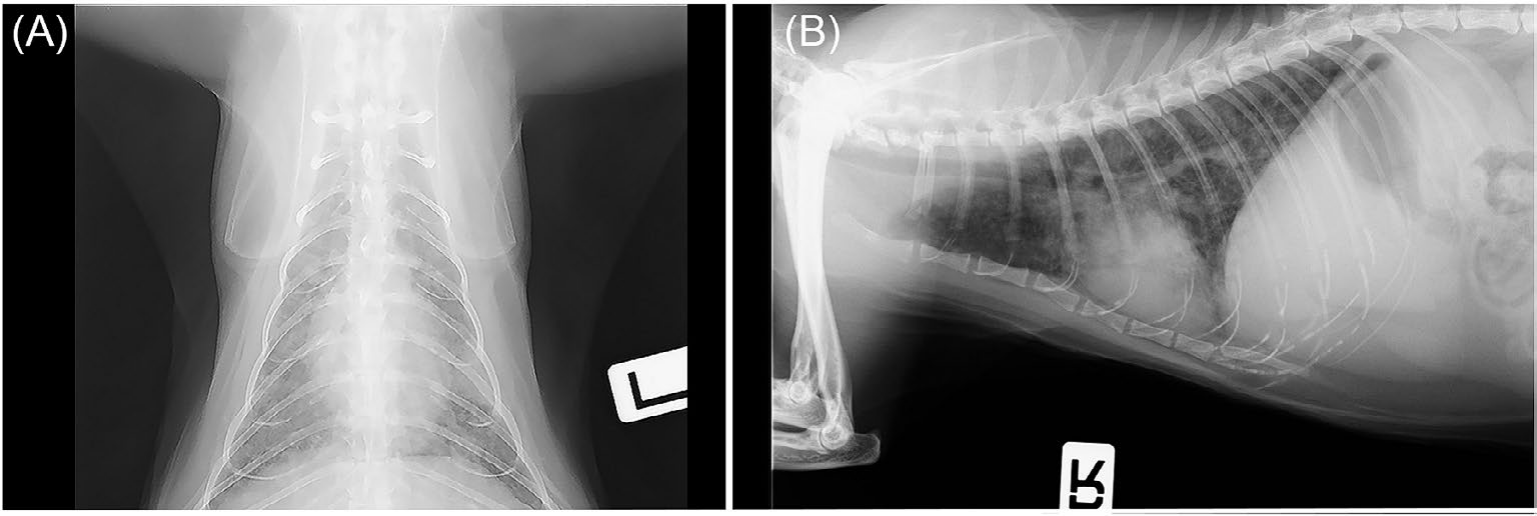
Thoracic radiographs of case 2. (A) Ventrodorsal view. (B) Left lateral view. Note the diffuse bronchointerstitial pattern with focal areas of increased soft tissue opacity.

The lung nodule aspirates contained moderate numbers of atypical cells admixed with occasional neutrophils, mast cells and lymphocytes in a background of moderate blood and frequent bare nuclei and cytoplasmic debris (Figure 5A,B). Atypical cells were mostly individualized and oval to spindle or stellate in shape, with a few forming small, poorly cohesive aggregates amid moderate light‐pink matrix material. Nuclei were round to occasionally bean‐shaped or indented, with reticular chromatin and 1–3 inconspicuous blue nucleoli. The cytoplasm was moderate, light blue, occasionally vacuolated, and had indistinct borders. Atypia was moderate and characterized by moderate anisocytosis and anisokaryosis, with occasional karyomegaly (Figure 5A,B). Mitotic figures were rare. Occasional phagocytosis of erythrocytes and platelets was observed. BALF cytology was highly cellular but had poor cell preservation. Intact cells resembled those in the lung nodule cytology and were admixed with few neutrophils, frequent ruptured goblet cells, and few small lymphocytes. The cytologic interpretation was consistent with neoplasia, likely of histiocytic origin. Given the imaging findings, FPLCH was suspected. HS and metastatic sarcoma or amelanotic melanoma could not be excluded.

**FIGURE 5:**
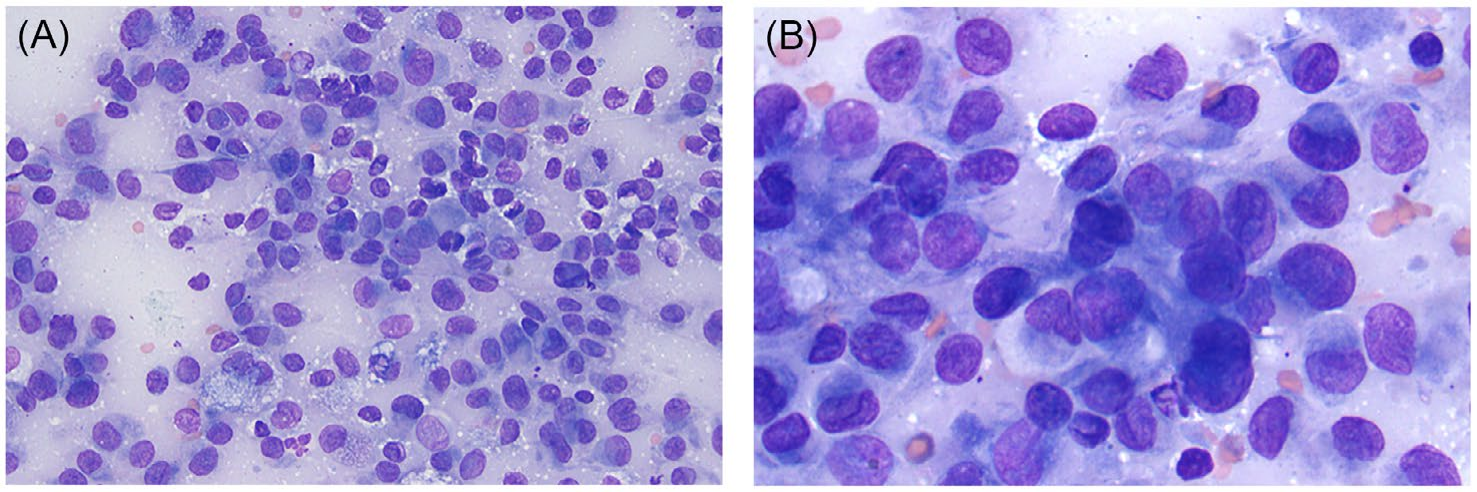
Fine‐needle aspiration cytology of lung nodules from case 2. (A) The neoplastic cells have moderate atypia. Occasional neutrophils and lymphocytes are in the background. Modified Wright's Stain. 40× objective. (B) Inconspicuous nucleoli can be appreciated in the atypical cell population. Modified Wright's Stain. 1000×.

Chemotherapeutics (i.e., lomustine) and palliative care were discussed. Given the poor prognosis associated with FPLCH and alternative differentials, humane euthanasia was elected.

A limited necropsy was performed after euthanasia. The lungs contained numerous multifocal to coalescing, variably sized, white to tan, raised firm nodules. Lung tissue samples were fixed in formalin and submitted for histopathology. The abdominal cavity was not examined.

Histopathological examination of H&E‐stained lung tissue revealed nodular, multifocal to coalescing hypercellular areas involving approximately 75% of the tissue, with partial loss of normal architecture (Figure 6A–C). Alveolar and bronchiolar lumina were filled with a densely cellular, infiltrative, non‐capsulated, and poorly demarcated population of atypical round cells forming small sheets. These cells had well‐defined borders and moderate atypia. Cells measured approximately 2–5 erythrocytes in diameter. Nuclei were eccentric, oval to reniform, with occasional additional indentations, coarsely clumped chromatin, and inconspicuous nucleoli. Cytoplasm was abundant, often with fine and occasionally larger empty vacuoles. There were ten mitotic figures in an area of 2.37 mm^2^. Clusters of these cells were also infiltrating the bronchial lumina, with scattered peribronchial lymphoid infiltrates.

**FIGURE 6:**
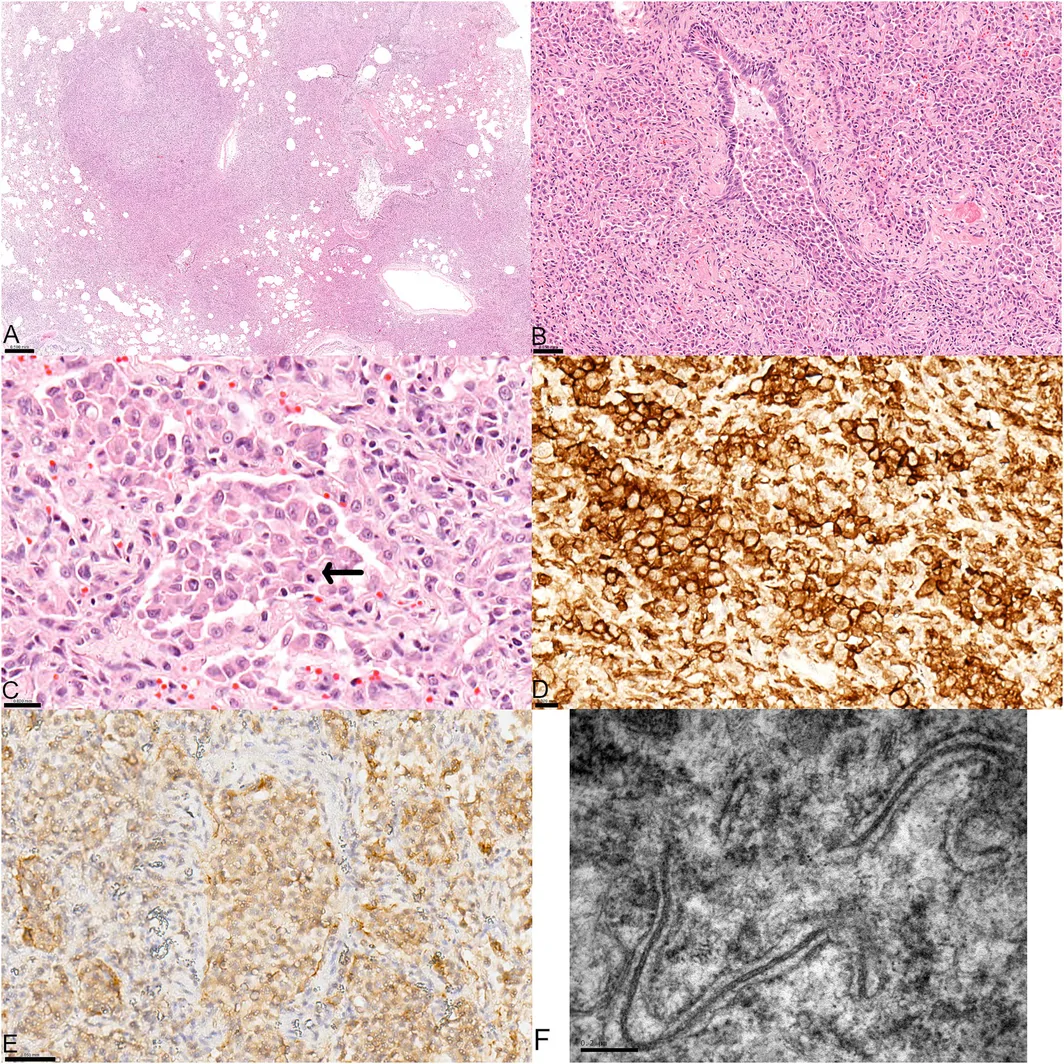
Histological, immunohistochemical, and ultrastructural features of case 2. (A) There are nodular, poorly demarcated, densely cellular, infiltrative neoplastic masses, with a peribronchial/peribronchiolar distribution. H&E. Bar = 0.5 mm. (B) The neoplastic cell population is composed of solid sheets of round cells filling alveolar spaces, plugging bronchioles (at the center of the image), and distorting the pulmonary architecture. H&E. Bar = 50 μm. (C) Neoplastic cells are round to oval and have a moderate amount of eosinophilic, occasionally vacuolated cytoplasm. Nuclei are oval to spindle with finely punctate chromatin and an occasionally small nucleolus. Anisocytosis and anisokaryosis are moderate, with rare mitotic figures (arrow). H&E. Bar = 20 μm. (D) The majority of the neoplastic population exhibits strong cytoplasmic and membranous labeling to IBA‐1. IBA‐1 immunohistochemistry (IHC). Hematoxylin counterstain. Bar = 20 μm. (E) Most neoplastic cells exhibit moderate cytoplasmic and membranous labeling to E‐cadherin. E‐cadherin IHC. Hematoxylin counterstain. Bar = 50 μm. (F) The neoplastic cells contain cytoplasmic, elongated, tortuous, multilaminar structures consistent with Birbeck granules. Transmission electron microscopy. Bar = 0.2 μm.

Due to the high suspicion for FPLCH, an immunohistochemistry panel including anti‐Iba1 and anti‐E‐cadherin was performed (Table S1). The atypical cells were diffusely positive for anti‐Iba1 and moderately to multifocally positive for anti‐E‐cadherin (Figure 6D,E).

Lung tissue was preserved in a solution of 2.5% glutaraldehyde in 100 mM phosphate buffer at pH 7.0 and submitted for transmission electron microscopy (TEM). Elongated, tortuous, multilaminar (penta‐laminar) structures with alternating electron‐dense and electron‐lucent layers within the neoplastic cells (Figure 6F) were consistent with Birbeck granules, confirming a diagnosis of FPLCH.

## 3 Discussion

To our knowledge, this is the first published detailed cytologic description of ultrasound‐guided FNA samples of subsequently confirmed FPLCH lung nodules. Previous reports have relied primarily on postmortem histologic evaluation, with only one case diagnosed antemortem via BALF cytology and immunocytochemistry [1, 2, 3, 4, 5, 6]. Our findings demonstrate that lung FNA samples can yield sufficient cellularity and morphologic detail to raise a strong suspicion of FPLCH, expanding the diagnostic options for this rare disease.

FPLCH primarily affects middle‐aged to older cats (typically 7–15 years old); a recent report described the disease in an African lion [1, 2, 3, 4, 5, 6, 7]. Clinical signs typically include lethargy, hyporexia, weight loss, and respiratory distress for days to months. In contrast to previously published cases, Case 2 did not present with dyspnea; pulmonary lesions were detected incidentally when performing thoracic radiographs to screen for metastasis of a suspected abdominal tumor. This suggests that FPLCH may have a chronic, slow evolution, with overt respiratory distress manifesting later in the disease.

In contrast with previous reports describing FNA of pulmonary parenchyma as non‐diagnostic due to low cell yield, the cases described here yielded highly cellular samples with features suggestive of malignancy, most likely of histiocytic origin, with FPLCH as the primary consideration [1, 3]. The degree of cytologic atypia, characterized by moderate anisocytosis and anisokaryosis, with rare karyomegaly, multinucleation, and mitotic figures, was considered less suggestive of HS, where these findings are typically more marked. Conversely, the atypia exceeded what is expected in inflammatory macrophagic infiltrates, where karyomegaly is usually not observed. Ziehl‐Neelsen staining was performed in Case 1 to specifically exclude mycobacteriosis. The absence of skin lesions excluded visceral involvement by feline progressive histiocytosis, and only rare phagocytosis of erythrocytes did not support hemophagocytic HS. Based on cytologic findings, FPLCH was the leading antemortem tentative diagnosis in both cases. Imaging findings were consistent with previously reported radiographic and CT abnormalities and excluded the presence of primary masses elsewhere that would support pulmonary metastasis [1, 2, 3, 4, 5, 7].

Post‐mortem histologic findings were similar to those previously reported cases [1, 2, 3]. Diagnostic confirmation of FPLCH typically involves immunohistochemistry (Table S2). The most extensive immunophenotypic profile described in FPLCH is E‐cadherin^+^/ Iba‐1^+^/CD1a^+^/CD5^+^/CD18^+^/Vimentin^+^/MHCII^+^/CD204^−^/CD3^−^/CD4^−^/CD20^−^/CD45R^−^/CD79a^−^ [1, 2, 3]. In this report, only Iba‐1 and E‐cadherin (Cases 1 and 2) and CD204 (Case 1) expressions were assessed. Nevertheless, the cytologic and histologic findings, combined with positive Iba1 and E‐cadherin expression, negative CD204 expression in Case 1, and TEM findings consistent with Birbeck granules in case 2, were considered sufficient to confirm FPLCH [3, 8, 9]. The CD204 positive cells admixed with the atypical cells in case 1 were interpreted as macrophages, which are expected to be positive while Langerhans histiocytes are negative [3]. The concurrent presence of macrophages admixed with neoplastic histiocytes may complicate the interpretation of cytology samples, especially in BALF samples, where alveolar macrophages may outnumber neoplastic histiocytes. In this report, case 1 was diagnosed with pyogranulomatous inflammation based on BALF, but the slides were unavailable for review at the time of FNA submission. In case 2, poor cellular preservation in the BALF slides limited evaluation.

Standardized treatment of FPLCH has not been established because of the rarity and severity of the disease. In humans, pulmonary Langerhans cell histiocytosis frequently involves MAPK‐pathways alterations [10]. Therefore, treatment with a TKI, as pursued in Case 1, may target similar mutations, although such mutations have not yet been documented in veterinary patients. Chemotherapy with the alkylating agent lomustine was discussed in Case 2, but the owners elected humane euthanasia.

Published data on prognosis are sparse, but outcomes are generally poor because of advanced pulmonary disease at diagnosis. Five reported cats either died or were euthanized within 24 h of presentation [1, 2, 3]. The longest documentation of respiratory signs before presentation is 24 weeks without treatment and 7 months with concurrent dexamethasone [1, 3]. The longest reported survival after presentation without treatment is 1 month, whereas cats receiving treatment have lived up to 2 months [1, 2]. Case 1 survived about 3 months after initial presentation with oral and inhaled steroids and a TKI, representing the longest survival documented in veterinary literature to date.

One limitation of this case report is the absence of a complete post‐mortem evaluation, which would have allowed further assessment of the extent of FPLCH and identification of additional comorbidities. Immunocytochemistry (including CD1a and E‐cadherin) would have been useful to achieve a confirmed ante‐mortem diagnosis of FPLH but was not performed. Unstained cytologic preparations were not available in our cases, and the reliability of the use of these markers on stained cytology samples is unknown.

In conclusion, this report provides a detailed description of the cytologic features of lung nodule FNA in FPLCH. Previously, transthoracic FNA of the lungs was reported to be of no diagnostic value because of low cellularity [1, 3]. Our cases demonstrate that diagnostic‐quality samples of FPLCH can be obtained via ultrasound‐guided FNA of pulmonary nodules. This expands the options for antemortem diagnosis, offering a less invasive alternative to bronchoscopy and BALF collection, which require general anesthesia, whereas transthoracic FNA can be performed under sedation.

## Disclosure

The authors have indicated that they have no affiliations or financial involvement with any organization or entity with a financial interest in, or in financial competition with, the subject matter or materials discussed in this article.

## Conflicts of Interest

The authors declare no conflicts of interest.

## Supporting information


**Table S1:** Details of antibodies used for confirming the diagnosis of two cases of Feline Pulmonary Langerhans Cell Histiocytosis.

**Table S2:** Summary of the discussed antibodies utilized for immunohistochemistry, the target cell population and documented staining characteristics of the histiocytic diseases observed in cats [3, 4, 7, 11, 12, 13, 14].

## Data Availability

The data that support the findings of this study are available from the corresponding author upon reasonable request.
