# Supplementary material for: Fine‐Needle Aspirate Cytology of Feline Pulmonary Langerhans Cell Histiocytosis in Two Cats
Source: Vet Clin Pathol. 2026 Aug 14;55(3):438–44. doi: 10.1111/vcp.70141 (PMC13730990; doi:10.1111/vcp.70141)
Supplement: Supplementary file 1 [file VCP-55-438-s001.docx]

Supplementary Table 1. Details of antibodies used for confirming the diagnosis of two cases of Feline Pulmonary Langerhans Cell Histiocytosis

|  | Antibody | Counter stain | Host | Clonality | Clone | Supplier | Dilution |
| --- | --- | --- | --- | --- | --- | --- | --- |
| Case 1 | **Iba1**^1^ | Hematoxylin | Rabbit | Polyclonal | N/A | Wako | 1:2500 |
|  | **CD204**^1^ | Hematoxylin | Mouse | Monoclonal | SRA-E5 | TransGenic | 1:800 |
|  | **E-cadherin** ^1^ | Hematoxylin | Mouse | Monoclonal | NCH-38 | Dako | 1:50 |
| Case 2 | **Iba1**^2^ | Hematoxylin | Rabbit | Monoclonal | EPR16588 | Abcam | 1:500 |
|  | **E-cadherin** ^2^ | Hematoxylin | Mouse | Monoclonal | NCH-38 | Dako | 1:250 |

**Legend**: N/A not applicable. ^1^ Antigen-antibody reaction visualized using Dako EnVision+ System-HRP labelled polymer secondary anti-mouse/rabbit and DAB substrate chromogen system. ^2^ Antigen-antibody reaction visualized using EnVision FLEX, HRP polymer secondary anti-mouse/rabbit and DAB substrate chromogen system.
