# Supplementary material for: Fine‐Needle Aspirate Cytology of Feline Pulmonary Langerhans Cell Histiocytosis in Two Cats
Source: Vet Clin Pathol. 2026 Aug 14;55(3):438–44. doi: 10.1111/vcp.70141 (PMC13730990; doi:10.1111/vcp.70141)
Supplement: Supplementary file 2 [file VCP-55-438-s002.docx]

Supplementary Table 2. Summary of the discussed antibodies utilized for immunohistochemistry, the target cell population and documented staining characteristics of the histiocytic diseases observed in cats. ^3, 4, 7, 11, 12^

| **Antibody** | **Target cell** | **Feline pulmonary Langerhans cell histiocytosis** | **Histiocytic Sarcoma** | **Hemophagocytic histiocytic sarcoma** | **Feline progressive histiocytosis** | **Supplier^3^** |
| --- | --- | --- | --- | --- | --- | --- |
| **CD1 a** | Dendritic cells, sub-population of B-cells and activated monocytes macrophages, thymocytes | +^3, 4,7^ | +^4, 7, 11^ | -^4, 11^ | +^4, 7, 11^ | LABL  , UC Davis, CA, USA |
| **CD4** | T helper cells, activated macrophages/ monocytes and interstitial dendritic cells, canine neutrophils | -^3^ | N/A | N/A | N/A | LABL  , UC Davis, CA, USA |
| **CD5** | T cells  (most, not all), subset of B-cells, subset of dendritic cells | +^3^ | N/A | N/A | +/-^5, 7, 12^ | LABL  , UC Davis, CA, USA |
| **CD18** | All leukocytes | +^3, 4, 7^ | +^4, 7, 11^ | +^4, 11^ | +^4, 7, 12^ | LABL  , UC Davis, CA, USA |
| **CD204** | Tissue-resident macrophages | -^3, 4^ | +/-^4^ | N/A in cats^4^ | +/-^4,12^ | TransGenic, Kobe, Japan |
| **E-cadherin** | Langerhans cells, Epithelia, some leukocytes | + ^3, 4, 7^ | N/A | N/a | -^4, 12^ | BD Transduction Laboratories, San Jose, CA, USA |
| **Iba-1** | Monocytic/histiocytic/dendritic  cells | +^3, 4^ | +^4^ | +^4^ | +^4^ | FujiFilm Wako Chemicals, VA, USA |
| **MHC II** | Lymphocytes (B and T cells), Dendritic cells, Macrophages | +^3^ | +^4, 11^ | +^11^ | +^4, 12^ | LABL, UC Davis, CA, USA |

Legend: + positive; – negative; ± variable or inconsistent; N/A not assessed. Superscripts refer to source references below.
